# Novel Use of a Shoulder Spica: Treatment for Midshaft Humerus Fracture in the Presence of Limb Deficiency

**DOI:** 10.7759/cureus.31169

**Published:** 2022-11-06

**Authors:** Elizabeth M Benson, Timothy Torrez, Chibuike Obinwa, Michael Conklin

**Affiliations:** 1 Department of Orthopaedic Surgery, University of Alabama at Birmingham, Birmingham, USA; 2 Department of Orthopaedic Surgery, University of Utah, Salt Lake City, USA

**Keywords:** upper extremity casting, congenital limb deficiency, shoulder spica cast, greenstick fracture, midshaft humerus fractures

## Abstract

Pediatric midshaft humerus fractures are typically managed with a hanging arm cast, Sarmiento bracing, coaptation splint, or a combination of these treatment options. Here we report a novel use of a shoulder spica cast in the treatment of a midshaft humerus fracture in the presence of limb deficiency. Current treatments proved unsuccessful in maintaining adequate alignment, specifically the varus deformity of the fracture. A shoulder spica was able to successfully maintain acceptable alignment throughout the duration of the patient's healing process. This nontraditional use of a shoulder spica cast shows the practicality of its ability to be utilized for the treatment of unique upper extremity orthopedic obstacles.

## Introduction

Congenital reduction deficiencies of the upper extremities are estimated to affect 5.25 per 10,000 live births annually [1]. Amniotic band syndrome is a congenital disorder associated with the in-utero formation of fibrous bands that risk fetal entanglement and constriction of the vasculature, and in severe cases can result in autoamputation [2]. There are many studies available highlighting obstacles that children with upper extremity limb deficiencies face [2,3,4]. However, even with limitations imposed by their congenital syndrome, many of these children can go on to participate in normal childhood activities which expose them to common musculoskeletal injuries [5,6]. 

Humeral shaft fractures comprise about 3% of pediatric fractures and most can be managed nonoperatively due to the robust remodeling process in children [7,8]. Non-operative options include a hanging arm cast, coaptation splint, Sarmiento brace, or sling and swathe [7]. In most settings, given the soft tissue overlay of the humerus, a mild to moderate amount of angulation is not visible and does not necessitate surgical correction unless the patient is approaching skeletal maturity [9]. 

The current case is unique in that it focuses on the treatment course of a five-year-old male with a history of amniotic band syndrome that presented with a humeral shaft greenstick fracture after a fall onto an outstretched hand. Due to the congenital limb deficiency just below the elbow, there was not an adequate amount of forearm on which to suspend a hanging arm cast, coaptation splint, or Sarmiento brace.

## Case presentation

**Figure 1:**
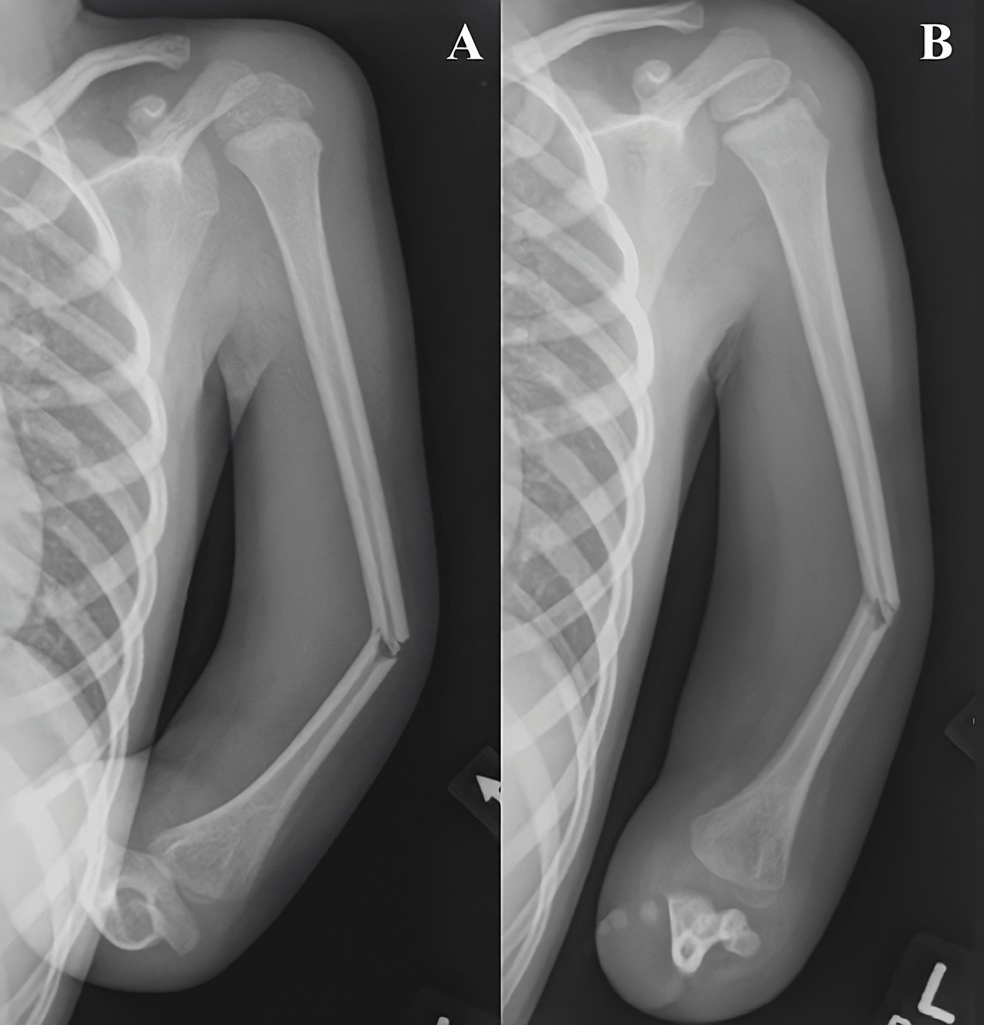
Initial imaging showing varus angulation of the left midshaft humerus fracture on oblique (A) and anteroposterior (AP) (B) views.

A five-year-old male presented to the ED with a left humeral shaft greenstick fracture. The patient fell while riding on a bike and landed on his outstretched left arm. On physical examination, there was swelling with no signs of an open fracture. The fracture occurred proximal to a transverse limb deficiency 2 cm distal to the elbow caused by congenital amniotic bands. Radiographs at the time of admission disclosed significant varus angulation (Figure 1). The patient was discharged with a Sarmiento brace and told to follow up in the orthopedic clinic. 

**Figure 2:**
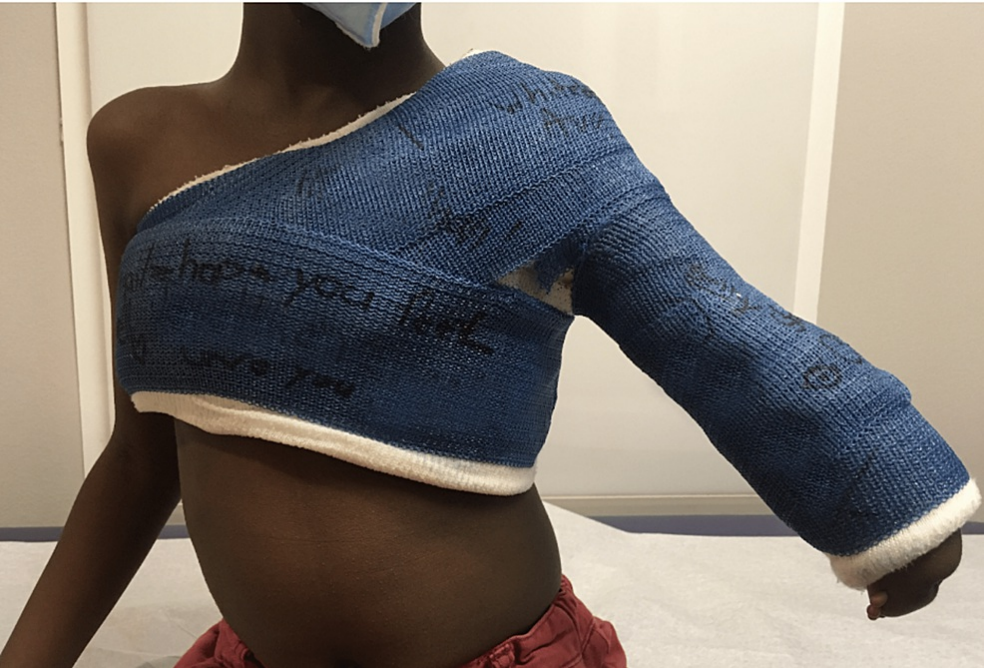
This image shows the shoulder spica cast applied following reduction of the patient’s fracture.

**Figure 3:**
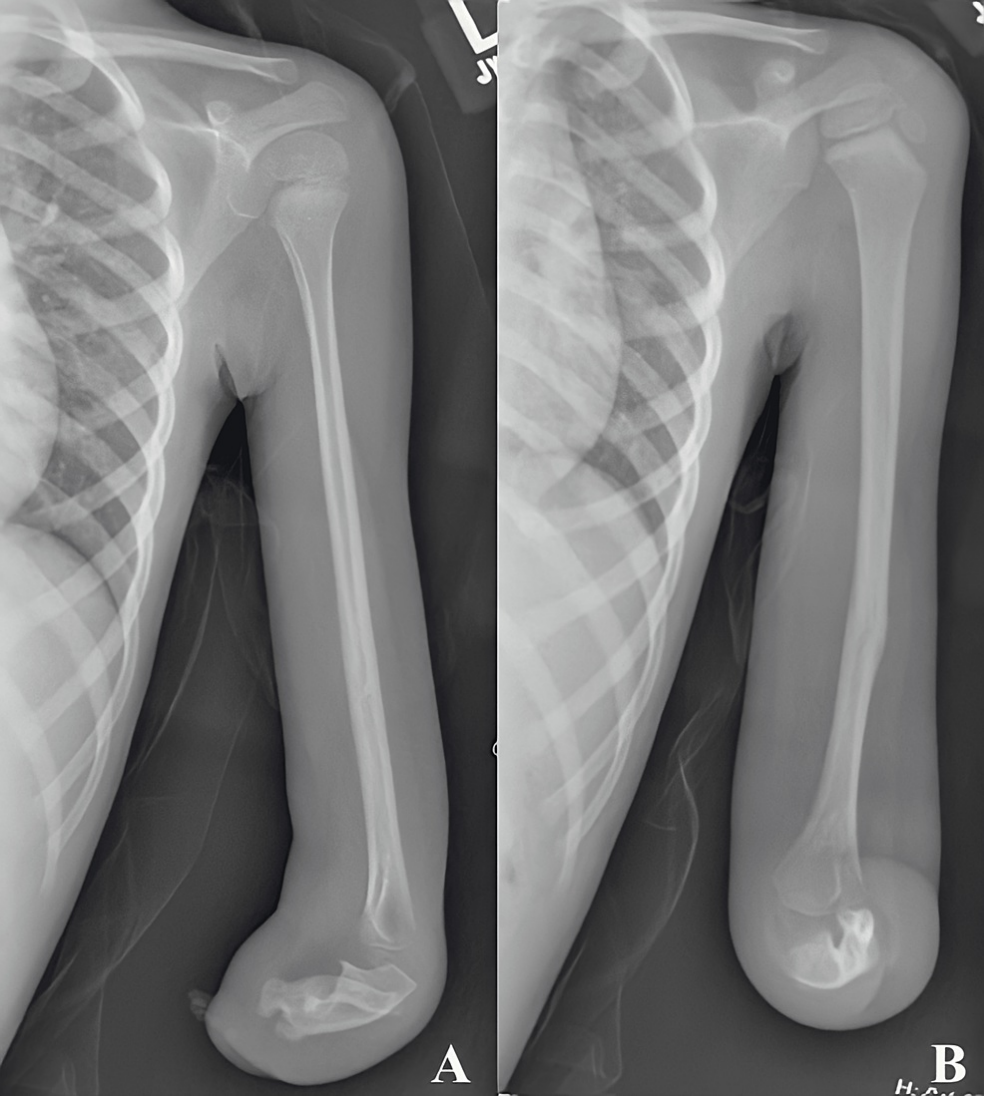
Imaging obtained seven weeks post-injury demonstrating appropriate alignment and fracture healing on lateral (A) and anteroposterior (AP) (B) views.

On follow-up in the orthopedic clinic, radiographs showed no improvement in alignment with 40 degrees of varus angulation. Based on this persistent fracture malalignment, the treating orthopedic surgeon determined this fracture to require manipulative reduction. After informed consent, the patient was transferred to the ED where closed reduction under IV sedation was accomplished. A shoulder spica cast was applied due to anticipated difficulties suspending a cast or splint (Figure 2). The arm was casted in abduction and was maintained at this angle by reinforcing the connection of the appendicular cast to the axial portion on the cast. He was discharged home with cast care instructions to keep the cast clean and dry. Two-week follow-up radiographs disclosed maintenance of reduction, and at four weeks, X-rays out of the cast disclosed excellent alignment with appropriate callus. The patient was placed in a Sarmiento brace. At seven-weeks post-injury, the patient had full range of motion, and radiographs showed adequate healing with good alignment (Figure 3). The patient was discharged.

## Discussion

While congenital limb deficiencies can present initial orthopedic obstacles, 50% of upper limb amputees experience additional challenges in their remaining extremity including orthopedic conditions such as fractures [10].

This case presented the unique challenge of devising treatment for an angulated greenstick diaphyseal humerus fracture in a congenitally amputated limb for which traditional treatment failed. Traditional treatments such as coaptation splints, hanging arm casts, and Sarmiento braces are widely accepted throughout the literature and utilized in clinical practice due to their ability to appropriately and conservatively manage pediatric midshaft humerus fractures [11]. However, all require some ability to suspend the device on the extremity which was problematic in this case. Though one could argue that an aggressive supracondylar mold may have been used to suspend a long arm cast, the shoulder spica added the benefit of being able to reliably correct varus by abducting the arm.

Acceptable angulation of pediatric humeral shaft fractures must be equal to or less than 25 degrees for proximal diaphyseal fractures and 15 degrees for distal diaphyseal fractures [7]. Due to the patient’s persistent angulation of 40 degrees, formal sedation and reduction were necessary to achieve acceptable alignment with the placement of a shoulder spica cast to add abduction to maintain appropriate reduction of the varus deformity. 

Very little data is available for indications and uses of a shoulder spica. Historically the technique has been described in articles dating back to the 1920s as a means to supply an abduction orthotic in the management of humeral and shoulder pathology [12]. One of the most detailed uses of a shoulder spica is by Shah et al., who outlined the use of shoulder spica casts in neonates undergoing shoulder muscle transfers [13]. They challenged the need for expensive equipment in order to apply the cast and presented a cost-effective technique. Similar to their technique, the shoulder spica cast applied was done so in a cost-effective manner using limited special materials. Caution was taken to provide room for chest expansion. Other uses for shoulder spica casts include hemivertebrae excision and brachial plexus surgeries including intervention for obstetric brachial plexus palsy [13,14,15]. A shoulder spica cast has also been used along with surgical intervention for a proximal humerus fracture with associated shoulder dislocation [16].

## Conclusions

In conclusion, our case demonstrates the challenges of treating patients with orthopedic concerns in the remaining limb of congenital limb deficiencies. While not commonly employed, we feel that the current case illustrates the value of shoulder spica casting in managing an unusual combination of problems in the upper extremity.
